# The Impact and Challenges of Education and Administration in VET on Economic Growth in Oman During the COVID-19 Period

**DOI:** 10.3389/fpsyg.2022.794239

**Published:** 2022-05-16

**Authors:** Amna Alzadjali, Fahriye Altinay, Gokmen Dagli

**Affiliations:** ^1^Department of Educational Administration and Supervision, Near East University, Nicosia, Cyprus; ^2^Department of Educational Administration, Near East University, Nicosia, Cyprus

**Keywords:** educational administration, vocational education training, employment opportunity, economic growth, COVID-19

## Abstract

The COVID-19 pandemic is still a major global health problem that had substantial consequences on people’s daily lives. This paper evaluates the impact of education and institutional management on Vocational Education and Training (VET) schools in Oman during the COVID-19 period. The purpose of this study is to understand the impact of the COVID-19 pandemic and identify possible challenges that may affect its impact on economic growth. This qualitative research is used as the main methodology of the study. Qualitative data are collected through convenience sampling of 108 VET college students and staff using interviews and questionnaires. The study revealed that the administration plays an important role in economic growth. The students suggested that the national educational administration of the government of Oman plays an important role in their education and that this in turn churns out industry-ready individuals who will impact the economic growth. The government’s role, especially financially post-pandemic lockdown, will be a critical determinant of VET’s impact on economic growth. This study introduces a new perspective on education administration from the perspective of students and staff of VET colleges.

## Introduction

When the COVID-19 pandemic broke out, people worldwide realized how important it was to keep epidemics under control. COVID-19 has brought many challenges in both schools and public places, such as in market places as well as in workplaces (Slimi, 2020). People’s behavior and personality have been affected greatly. Student behaviors also changed. Everyone must wear a mask to avoid the spread of the coronavirus. In some countries, a person can be arrested for not putting on a mask. As a measure to curb the spread of the virus, certain precautions were introduced and are enforced by law enforcement agents. COVID-19 has also affected the behavior of the individual; it has made people change their behavior, conduct, and attitude. This can be seen by how people maintain social distance in public places, regular sanitization of hands, as well as administering vaccines regardless of religion or tradition (Al Mahdouri and Al Saidi, 2020). Coronavirus has impacted immensely on socially, economically, as well as religiously. As a stern measure to curb the spread of the coronavirus, congregants were not allowed to converge in great numbers in their mosques and churches. In some instances, they were not allowed to gather at all as some communities were in total lockdown. Gathering of more than 50 people was not allowed, and in this way, the social life of people was affected.

Some countries, such as France, Italy, the United States of America, the United Kingdom, and Oman, as well as most of the African states, have lost many citizens as a result of COVID-19. Many families have lost their beloved ones due to the coronavirus. The devastating effect of the coronavirus has gone to an extent that some families have lost all parents, and in some cases, the whole family. Family members with underlining health conditions such as respiratory problems and heart problems were heavily hit by the virus if great precaution was not exercised. Those who were affected were of the economic age group (the front runners), as they were exposed to the virus. The economic age group contributes greatly to national economic growth (Al Mahdouri and Al Saidi, 2020; Slimi, 2020; Ceesay, 2021; Chumpitaz-Carrillo and Sevillano-Jimenez, 2021; Fowler et al., 2021).

COVID-19 further changed students’ behavior as face-to-face learning was abandoned and an innovation was introduced. Murphy (2020) says that online learning, which replaced face-to-face teaching, has also brought its challenges. Online learning requires gadgets such as smartphones, iPads, and laptops from the learner. All these gadgets have to be bought by parents and guardians which calls for extra money for the education of the child. Data have to be provided to students for them to be always connected to their lecturers. All these are some of the challenges brought by COVID-19. As a measure to mitigate the transmission of COVID-19, certain precautions were put into place. These measures include total closure of schools, suspension of classes in colleges, universities, all academic institutions, and a restriction on traveling (intercity travel), which in summary was called total lockdown. This was announced by Oman Supreme Committee on COVID-19. As a result of this announcement, the majority of schools and higher learning institutions adjusted their programs to go by the stipulations of the COVID-19 regulations (Kothaneth, 2020).

Vocational Education and Training will play an important role in helping revive economies after the pandemic lockdown by providing the training and education needed for an industry-ready workforce (Pilcher and Hurley, 2020).

The data for this study was collected from 108 students using convenience sampling of college students and staff. The study uses a random convenience sampling technique with a few inclusion and exclusion criteria. Inclusion criteria include students who have been in VET colleges for a least one full academic semester, fully employed academic staff who have been working on the campus for at least one academic semester, non-academic staff who work within the campuses, and institutional management staff who have been fully employed for at least one academic semester. Exclusion criteria included participants conducting any current research related to the impact of COVID-19 in different contexts and respondents who have been in VET for less than one academic semester. The study was carried out face-to-face at the VET colleges. The researcher ensured not to interrupt any educational activities and only willing participants in their free time were asked to participate. The Universities’ registrars were preinformed about the scope of the study before it was carried out.

### Literature Review

Many investigations into the effects of education on COVID-19 were conducted during the epidemic years. For example, Albaadani and Abbas (2020) studied the impact of COVID-19 on education in Yemen. Schools and universities in Yemen suspended their activities as a precautionary measure to prevent the spread of the new coronavirus among their students. As a result of this procedure, higher education institutions in Yemen are faced with a variety of challenges that make it difficult to quickly adapt to the new environment, including Internet and infrastructure weaknesses, difficulties dealing with change, academic career stability, university economic security, the complexity of some applied disciplines, student mental health, and the costs of rapid transformation. The study made some recommendations for overcoming this situation, as well as any other urgent situations that may arise in the future that could cause the educational system to shut down, including the establishment of a hybrid education system and the activation of an electronic portal for each university. Another investigation into Nepal’s educational system has been carried out to examine the effects and prospects of the COVID-19 epidemic (Pal et al., 2021). The purpose of this study is to shed light on the negative effects of the pandemic on Nepal’s educational system. According to the findings, the academic community in Nepal has been struggling due to a lack of suitable and acceptable online system infrastructure and qualified human resources. In addition, limited access to the Internet in rural and isolated places makes virtual academic activities difficult (Abumalloh et al., 2021). In the wake of the coronavirus pandemic (COVID-19), virtual and remote laboratories will play an increasingly important role in classroom instruction. Using a poll of students at Imam Abdulrahman Bin Faisal University, this study investigates the projected benefits of e-learning during the COVID-19 epidemic. It provides a new approach to investigating this topic. In total, 179 usable responses were subjected to Partial Least Squares Structural Equation Modeling (PLS-SEM). To test the hypothesis of Push-Pull-Mooring, this research looked at how learners’ responses changed when they were placed in virtual or remote educational environments.

Liu and You-Hsien Lin (2021) looked into how COVID-19 changed medical school education in Taiwan. An outbreak (COVID-19) has now been declared a global pandemic emergency. Although medical education has a significant impact on health care, little is known about those effects. This brief communication focuses on medical schools in Taiwan’s response to COVID-19 and how medical education has influenced that response. Ng (2021) conducted a study on the impact of the COVID-19 pandemic on preregistration medical radiation science education. According to the study’s goal, preregistration medical radiation science (M.R.S.) (medical imaging and radiation treatment) education has changed because of the COVID-19 pandemic. After the pandemic, preregistration M.R.S. education curriculum adjustments were made, and the consequences of such adaptations on stakeholders were examined in this literature review. The possible influence of the COVID-19 outbreak on education, staff development, and training in Africa was studied by Ceesay (2021) as well. The main goal is to understand how the COVID-19 epidemic affects education in Africa, particularly staff development and training.

Gui et al. (2021) studied the impact of COVID-19 on the energy use of higher education buildings and the implications for future studies on educational building energy use. This research was carried out to reveal changes in COVID-19 energy use and establish the related facilities management techniques for future forms of learning and teaching delivery on virtual campuses. When the COVID-19 academic year (February 17, 2020, to February 21, 2021) and a typical academic year (February 18, 2019, to February 16, 2020) of Griffith University, located in Southeast Queensland, Australia, were compared, the data from 122 buildings across five campuses were collected by PI Vision Platform and used to compare energy use using the *t*-test and multiple linear regression. The spring 2020–winter 2020 impact of the COVID-19 pandemic on surgical trainee education and well-being has been done by Ellison et al. (2021). This pandemic’s impact on surgical education and learner well-being is unknown. General surgery and surgical specialties check-in surveys were issued to Surgery Program Directors and Department Chairs in the summer and winter of 2020. They were then compared to a survey from the spring of 2020. There were statistical correlations found between items related to the self-reported ACGME Stage and the period studied.

The impact of COVID-19 on Indian schooling was examined in a study conducted. Dhanalakshmi et al. (2021) talk about the negative effects and challenges of climate change, but they also try to come up with solutions that could be used in other studies.

Following a year of the COVID-19 outbreak in Bangladesh, the impact of online education on university students’ anxiety and psychological well-being was studied. As a result of the devastation caused by coronavirus disease, extreme anxiety about the academic delay (FAD) and psychological anguish have emerged as major public health concerns worldwide (COVID-19). After 1 year since the devastating COVID-19 outbreak hit Bangladesh, researchers wanted to see the effect on current university students’ FAD and psychological stress symptoms of continued online education (Hossain et al., 2021).

However, on another note, in recent times (here and now), the slowness in economic growth has been necessitated by the advent of the COVID-19 crisis that created havoc in areas of production. Many industries have been closed down due to COVID-19, which directly affected production. Thousands of workers have been killed by COVID-19, among whom some of them were specialists, qualified personnel, directors of companies, skilled laborers, and even semi-skilled. All these have brought and are still imposing big challenges on the economies of nations because production is not performing at its maximum level.

Al Mahdouri and Al Saidi (2021) examined how behavioral science has contributed to shaping public policies during the COVID-19 pandemic, with a focus on the Sultanate of Oman. The outbreak of COVID-19 led to the spread of a massive global health pandemic that caused a surge in public policies adopted around the world, most notably policies related to health, travel, education, work, and freedom of movement. On the other hand, Slimi (2020) shed light on the experience of online learning and teaching in Oman in higher institutions under confinement circumstances. The paper targeted three main goals. First, the researcher sought to study the challenges of learning and teaching online in Oman. Second, to identify the opportunities offered by learning and teaching online, and third, to recommend solutions for better learning and teaching processes. Her findings revealed that although online learning and teaching are novel experiences in Oman, they are challenging experiences that have reinforced new styles of learning and teaching. Rachmadtullah et al. (2020) conducted a study that aimed to find out how effectively the use of the blended-learning learning model with Moodle applications in elementary school teacher education prepared students during the COVID-19 pandemic. This type of research has a quasi-experimental design with a pretest-posttest control group. When elementary school teachers are learning how to teach students during the COVID-19 pandemic, they can use the MOODLE blended-learning learning model. This model can be used as a network-based learning solution, as well as online.

Most previous research was done in countries with better resources and facilities than Oman, and only a few studies were done on health services, medical problems, public knowledge about COVID-19, teaching during the COVID-19 pandemic, and students’ satisfaction and achievement during the COVID-19 pandemic (Chumpitaz-Carrillo and Sevillano-Jimenez, 2021; Fowler et al., 2021; Quesada-Rodríguez et al., 2021). There was also very few research done to investigate the impact of the COVID-19 pandemic on students’ learning in Oman’s educational system. So, the goal of this study is to figure out how VET education and administration affect Oman’s economy and to look for problems that might happen during the COVID-19 period, which is when this study is done.

## Method

The literature review highlights critical challenges and drastic changes in the educational systems during and after COVID-19. To find out how this pandemic has affected VET education and administration in Oman, this paper will look at how these problems have affected the country’s economy.

This qualitative research is used as the main methodology of the study. Qualitative data are collected through convenience sampling of 108 VET college students and staff using interviews and questionnaires.

Government VET colleges across Oman were visited for the study after permission was sought from the relevant authority. In each college the researcher visited, permission was given by the registrar to have access to talk to the students in randomly selected departments. For the sake of this study, only two departments per college were worked with as a representation of the whole college, because it was not possible to work with the whole institution. The researcher wrote numbers 1 to 12, which were picked randomly by student representatives from the 12 departments in each college. Students who chose numbers one through two were automatically enrolled in the process. Volunteers from each department were worked with for data collection. The researcher was allocated a room in every college where the students were answering questions from the questionnaires. The researcher administered the questionnaires herself. This was important because the researcher could clarify any problems raised by participants. After this process, the researcher collected the questionnaires for data analysis and presentation. For the academic and non-academic staff, data were collected using focus group discussions as this was seen as befitting because they could discuss openly without any fear. The researcher sought permission from the registrar of every college to discuss the topics with the academic, non-academic, and institutional management staff who were willing to be involved in the focus group discussions. Gender and age of both the staff and students are shown in Table 2. Data were collected using focus group discussions. The researcher chaired the discussions. The demographical information for the participants is presented below in Table 1.

**TABLE 1:** Distribution of study respondents by occupation.

	Students	Academic Staff	Non-academic Staff	Institutional management Staff	Total students and staff at VET
Seib	10	1	1	1	1,373
Saham	9	2	1	1	645
Sur	11	1	1	1	568
Abri	9	1	1	1	538
Shinas	8	2	1	1	386
Al Buraimi	12	1	1	1	421
Al Khaboura	13	2	1	1	377
Salalah	9	1	1	1	1,086
Total	80	11	9	8	5,394
Percentage	74%	10%	8%	7%	90% confidence

*Source (Ministry of Manpower, 2020).*

**TABLE 2:** Distribution of respondents based on gender and age.

Variables	Variable description	Frequency	Percentage
**Students**			
Gender	Male	42	52.5 %
	Female	38	47.5 %
	Total	80	100 %
Age	17—20	56	70 %
	20-24	24	30%
	Total	80	100%
**Academic Staff, Non-Academic Staff and Institutional Management**			
Gender	Men	18	63.3 %
	Women	10	35.7%
	Total	28	100 %
Age	24—35	16	57%
	36—50	12	43%
	Total	28	100%

From Table 1, it is observed that the number of students is 80 which is 74% of the respondents, 10% of the respondents are academic staff, 8% of the respondents are non-academic staff, while 7% are institutional management staff. The respondents were sampled using the Taro Yamane (1967) sampling technique to get the minimum required respondents for the study using a 90% confidence interval.


$$n=\frac{N}{1+N\left(e^{2}\right)}\left(1\right)$$

Where *n* is the sample size, *N* is the population, and e is the margin of error. The confidence level was set to 90% making *n* = 98.17983254. The number of respondents for the study was 108 students and staff of VET.

Table 2 shows the gender and age distribution of the students and staff: 52.5% of the students from the VET school were male, while 47.5% were female; 70% were between the ages of 17 and 20 years, while 30% were between 20 and 24 years. For the staff, however, 63.3% were male, while 35.7% were female; 57% of them were between the ages of 24 and 35 years, while 43% of them were between the ages of 36 and 50 years.

### Data Collection Tools

The study uses focus groups and interviews with the students. The benefit of this preferred technique was its explicit use of group interaction to produce data and insights that would be less accessible without interaction. The researcher further identified that focus group discussion was suitable for this research because it works very well with a qualitative approach to obtain a deep understanding of social phenomena. This method collects data from a randomly selected sample of individuals rather than a statistically representative sample of the entire population. During the discussions, the researcher moderated the process to allow order to prevail. The researcher did not have any problems because all of the staff worked together very well during the discussions.

The study questions asked during the interviews:

1. *Give one example of how education has benefited the national economy.*
2. *Why did you prefer to be enrolled in VET college rather than other colleges?*
3. *Do education and administration greatly impact national economic growth?*
4. *Discussions on various types of education in general and in particular on Oman’s education system.*
5. *A brief analysis of the current education system in Oman in relation to the national economy.*
6. *A discussion on what must be done to improve the current education system.*
7. *A discussion on why VET is not like other systems of education.*
8. *A discussion on improvements they recommend their immediate institutional management should implement to enhance quality products.*
9. *A discussion on what the academic staff wishes their government to do to improve the current working conditions.*
10. *Are the online lectures effective for everyone?*
11. *Does the Ministry of Higher Education design effective software for e-learning?*
12. *Do the faculty and lecturers support the students and monitor their learning progress in emergency cases like COVID-19?*

## Results

In the study, the VET educational administration was found to be critical to economic growth. Many students opined that the national government should spend heavily on the country’s education system, from elementary to post-secondary levels. Education is almost always a failure if the government does not provide funds to improve it. The administration should also give a national vision in several ministry sectors, according to students, to fulfill its objectives.

### Administration on Economic Growth in Oman

From the focus group discussions conducted, it was revealed that the education administration plays a pivotal role in economic growth. Several students raised the important point that the national educational administration should invest a lot of capital in the education system, from primary to tertiary. If the government does not make available funds to boost the education sector, the outcome of education is normally a disaster. Students raised the idea that the administration should provide a national vision in various sectors of its ministries to meet its goals. The administration is divided into two: the national administration, which is the central government, and the educational administration, which is the head of the institution. The academic staff pointed out that the national administration (government) should coordinate the education system to achieve national goals. About 60% of the respondents opined that policies should be formulated, crafting strategies and programs that enhance the achievement of the national vision. There is a need for the government to revisit its national policies to remain viable and contemporary with what is happening globally. The government must make available funds for research, workshops, in-service courses, and conferences for the employees of various VET institutions to enhance performance. To support this, the World Bank (2008) cites Oman as one of the developing countries with rapid economic growth in a short span of time. The countries in this category are China, Japan, Botswana, Malaysia, Brazil, Indonesia, South Korea, Hong Kong, Thailand, Taiwan, and Vietnam.

The administration in various VET colleges should work closely with the national administration (government). Administration refers to several people, usually in charge of an institution. Their specific role is to coordinate and regulate all the activities taking place within the institution. The academic staff pointed out several points concerning their administration. They state that the administration must monitor, regulate, and moderate all activities around their colleges. They further explained that the administration should set the tone, culture, and strategies for the institution to realize its intended objectives as well as meet the goals of the national vision. It was also noted that the administration must have a proper structure, clear system, and excellent communication system among the institutional management, academic staff, students, and other non-academic staff to create a peaceful working environment. The students further raised the fact that the administration must regularly meet with students to hear their grievances. Meeting with the administration regularly helps to build good relationships between members of the institution, which will lead to better results that will help the country’s economy grow.

### The Impact of Education and Vocational Training on Economic Growth

From discussions conducted in the eight VET colleges, it was clear that there is a close relationship between education and economic growth. Many participants supported the view that education impacts greatly on the rate of economic growth in Oman. The participants, especially the academic staff, pointed out that the type of education determines the quality of skills acquired by individual citizens. The majority of the participants were of the view that a country that invests much in its education system and offers the right curriculum in its educational institutions, right from primary, secondary, and tertiary, in most cases, has a sound economy.

In their study, Hanushek and Wößmann (2007) stated that education quality is more important than educational attainment. Here, they are emphasizing the nature of the curriculum being offered. The curriculum must give a staff orientation to the students so that they appreciate careers that help boost the economy. In the discussion, it was said that VET has multiple benefits that can be classified into two groups: social benefits and economic benefits. On social benefits, we have crime reduction, individual encouragement, life satisfaction, and social integration (social fabric). On economic benefits, Oman has a labor market, a firm’s performance, employees’ productivity, employment opportunities, and professional and career development. All these benefits eventually contribute to the national economy. Much emphasis was also given to the government’s developing and rehabilitating VET institutions to produce quality graduates that are directly linked to the labor industry of Oman. Academic staff indicated that such basic knowledge of the subject area teaches the students the skills that are directly linked or related to various job opportunities. The world’s economy is changing each day, and as such, there is a need for vocational education training to change as well in their approach to meet new technological dynamics. This is supported by Benhabib and Spiegel (2005), who say that education facilitates the diffusion and transmission of knowledge needed to implement new technologies. In this regard, it is a common scenario that normal human capital can be enhanced through massive investment and a strong commitment to human factors such as education and training. It was pointed out that vocational education training enrolments have now, over the years, been consistently higher than those in universities in Oman. This is an indication that there is more demand for the workforce in the job market, a sign of an expanding economy. Whether directly or indirectly, vocational education training is an important component of the economy, whether in the private or public sectors. It offers work-based training, which is crucial for national development.

## Discussion

There is a general view that if a country spends significantly on education normally, its people would be able to live a standard life and its economy would be better, which would eventually lead it to go through the three stages of development easily—the third world, second world, and first world, respectively. However, this view may not be correct since there are a lot of factors to be considered for the national economy to grow. The type of governance, education system, culture, and religion, to mention a few, would influence the economy of a country. Of the few given factors, a proper education system is the bedrock of development. Investing in education is one of the major factors that boost economic growth in highly developed countries by fostering labor and capital productivity and technological innovations that boost national production. If labor is productive, the national economy is automatically positively affected. A closer examination reveals that most third-world countries’ or poor states’ education policies are out of step with current global trends when compared to first-world nations. Similarly, Oman is prioritizing education as a key component in transforming its economy. Europe, the United States, Asia, and South Korea are growing relatively faster as there is high productivity. South Korea is investing heavily in its education, and as a result, its economy is growing fast. Studies by Scherer and Hue (1992) show that the United States and Europe spend about 3% of their total domestic product on executives who have a high level of technical education, and a lot of money is channeled toward research and development, which enhances the probability of innovations. These innovations have a lot of impact on economic growth; 60% of the academic staff were of this view. It shows that the staff have a clear conscience that investing in education, be it primary, secondary, tertiary, or technical colleges like (VET), is key to economic growth. However, one cannot just conclude that the citizens’ spending many years in school would influence national economic growth. It does not work like that. There is a lot to be done. The national administration (the government) has to invest a lot of funds to cater to various activities in the education sector, especially VET. Research has to be carried out to find out which policies to adopt to increase the rate of economic growth. It has to be strategically planned and implemented. Human capital has to be fully resourced. Infrastructure has to be modernized to cater to technological advancement. When all is put in place, the products from the colleges will adapt to the world’s dynamics. Benhabib and Spiegal (1994) argue that a more educated labor force would innovate faster than a less educated one. This is also reiterated by Lucas (1988) and Mankew et al. (1992), who observed that the accumulation of human capital could increase the productivity of other factors, thereby increasing economic growth. Societies that invest in their labor force education and improving the quality of their human capital will reap the benefits in the 21st century. With innovations such as information technology, and technological advancements in other industries, the importance of an educated workforce can be observed.

It was noted with great emphasis from both participants (the students and the VET staff) that the most effective way of utilizing public and national resources is to improve the production and efficiency of the labor force. This is only mostly achieved when a lot of resources are invested in education, vocational education training, and skills development. The million-dollar factor to speeding up their economic growth rate is to improve productivity in all sectors, especially in this era of fast economic and technological dynamics, for them to compete internationally.

In this era where technology and development are the order of the day, it is imperative that states, through their national governments, properly manage the human and physical resources of their economies. It was further raised by both groups (students and staff) that during periods such as the period of COVID-19, the government must swiftly avail funds for the provision of protective clothes for the VET staff. Also, necessary arrangements must be put in place as fast as possible to avoid loss of life. Both the students and the teachers applauded the Oman Supreme Committee of COVID-19 for the announcement of the suspension of classes as the continuation of face-to-face learning could enhance the spread of the COVID-19 virus. As an alternative to this problem, online learning was deployed. However, every innovation has its strengths and weaknesses. The introduction of online learning presented numerous challenges to students, faculty, and parents. It was pointed out that online learning requires a lot of money to procure the right and correct gear for both the staff and the students for it to be effective. Advanced technology has to be there in terms of smartphones, laptops, iPads, Wi-Fi, provision of the Internet, and also the assurance of a dependable source of power to avoid unnecessary disruption during lessons.

## COVID-19 Technology and VET Oman Education Challenges During COVID-19

This pandemic, as it is still in our midst, is calling for all nations to have a paradigm shift from their traditional way of learning in their education system to a digital and distance learning approach. However, this comes with its challenges, for instance, the cost of digital gadgets, the credibility of the material used, adherence to new rules and conformity to the new rules of the management, and the use of student personal data (Murphy, 2020). Thiele (2003) suggests that there is a need to assess these challenges on the quality of online course delivery. They should produce positive outcomes that contribute to the building of the national economy. The new learning style and the new technologies have to complement each other for the students to produce the desired results during the COVID-19 pandemic. This is consolidated by Richmond and Cummings (2005), when they say that one of the ways to accomplish effective delivery of an online course on learner outcomes is to have a learning style framework incorporated into the learning and teaching situation.

The introduction of e-learning in VET in Oman is not a new style of learning; however, its consolidation was accelerated during the COVID-19 pandemic. The government of Oman perceives e-learning positively and considers information and communications technology (ICT) as an important element of its education system to improve the quality of education. E-learning helps students in Oman access learning resources instead of forcing them to go to schools and universities (Musawi, 2002). Kothaneth (2020) adds that e-learning is not a new concept for higher education institutions in Oman and goes on to say that e-learning can help students ease COVID-19 risks.

Major challenges caused by COVID-19 are that students are distracted, teachers are not sufficiently trained, and technological infrastructure is far from fully ready to operate. All these conditions are forcing governments and service providers to be under pressure to adjust faster to ensure undisrupted learning in schools and universities during such difficult times as the COVID-19 period.

Both private and public institutions should provide and develop the skills needed to facilitate development, change, and growth to turn around the economy. The data obtained by this research reveal that Oman is competing very well with other states of the world in trying to adjust its national programs to meet the current economic challenges. This study shows that there is a positive and significant correlation between economic growth and vocational education training (VET) in Oman. The findings are parallel with the views of Easterlin (1981) who avers that vocational education training enhances technology diffusion, labor productivity, and economic growth.

## Conclusion

The results of this study show that qualitative comments are showing the link between economic growth and vocational education training. Training of human force is key to development because it imparts knowledge and skills that are essential in the turning around of the economy. Investing in education, directly or indirectly, is linked to development because it enhances the skills and knowledge of the labor force and promotes the maximum production of goods and services. Oman, over many years, has invested a lot of capital toward improving vocational training. The nature of the infrastructure, the quality of VET products, and the caliber of institutional management staff, academic, and non-academic staff are evidence of how the government of Oman is promoting its education system. The study revealed that there is evidence to support that there is a very clear positive relationship between economic growth and VET in Oman. It is noted that there is a great link between education and administration in the economic growth of Oman. It is further revealed that Oman has invested a lot in VET by providing capital and crafting favorable policies that promote vocational training, which produces skilled labor, which is a major component in turning around an economy or in economic transformation (economic growth). Countries such as America, Britain, Germany, and Oman, to mention a few, have since used VET to determine their economic growth. The study also revealed that COVID-19 has brought many challenges, and these challenges have negatively affected the economies of various nations. Total lockdowns and semi-lockdowns have reduced production in many industries across the globe, and Oman is not spared.

### Recommendations

Following the above conclusions, the following recommendations were made:

• The government should build more VET colleges to cater to more students as the enrolment in the current colleges is high and should also prepare a good platform for online learning.
• The Ministry of Higher Education, Research, and Innovation should carry out a constant evaluation of VET colleges for them to produce quality products that are skilled and effective in the job market and should introduce a software program that works with the Internet that will be free for all.
• The government, through relevant ministries, should make funds available for any improvement to VET colleges. For example, financing workshops, continuous provisional development courses for employees to enhance their skills and knowledge to meet world standards, and designing effective software for e-learning.
• Creating good working relations among employees (institutional management staff, academic staff, non-academic staff, and students).
• Introducing VET in the early stages of the education system, for example, at the primary level, would enhance specialization like what other developed nations are doing (United Kingdom, Germany).
• Improving the working conditions of employees through raises in salaries, giving them incentives.
• The policy implications of the study are that the exponential growth in vocational education training in Oman should be accompanied by suitable policies that enhance job creation across all walks of life.

## Data Availability Statement

The original contributions presented in the study are included in the article/supplementary material, further inquiries can be directed to the corresponding author/s.

## Ethics Statement

The studies involving human participants were reviewed and approved by Near East University Ethical Committee Board. The patients/participants provided their written informed consent to participate in this study.

## Author Contributions

AA, FA, and GD collectively conducted the study, contributed significantly to the research, wrote and revised the article. All authors approved the final version of this article.

## Conflict of Interest

The authors declare that the research was conducted in the absence of any commercial or financial relationships that could be construed as a potential conflict of interest.

## Publisher’s Note

All claims expressed in this article are solely those of the authors and do not necessarily represent those of their affiliated organizations, or those of the publisher, the editors and the reviewers. Any product that may be evaluated in this article, or claim that may be made by its manufacturer, is not guaranteed or endorsed by the publisher.
